# TBC1D25 Regulates Cardiac Remodeling Through TAK1 Signaling Pathway

**DOI:** 10.7150/ijbs.41130

**Published:** 2020-02-21

**Authors:** Sen Guo, Yuan Liu, Lu Gao, Fankai Xiao, Jihong Shen, Shiying Xing, Fan Yang, Wencai Zhang, Qiangwei Shi, Yan Li, Luosha Zhao

**Affiliations:** 1 Department of Cardiology, The First Affiliated Hospital of Zhengzhou University, No.1 Jianshe East Road, Zhengzhou, China; 2 Henan Key Laboratory for Esophageal Cancer Research, the First Affiliated Hospital of Zhengzhou University; 3 Department of Electrocardiogram, The Second Affiliated Hospital of Zhengzhou University, No.2 Jingba Road, Zhengzhou, China; 4 Department of Cardiology, The First Affiliated Hospital, and College of Clinical Medicine of Henan University of Science and Technology, Luoyang, China.

**Keywords:** cardiac remodeling, TBC1D25, TAK1, signaling pathway

## Abstract

Cardiac remodeling is a major early event of heart failure, which is regulated by multiple signaling pathways. Here, we demonstrate that TBC1D25 is upregulated during pathological cardiac remodeling. The aim of this study is to determine the role of TBC1D25 in cardiac remodeling and to illustrate the underlying molecular mechanism. Specifically, cardiac remodeling was induced in TBC1D25-KO mice and their wild-type control mice through partial transverse aortic constriction (TAC) of aortic arch. Knockout TBC1D25 exacerbated cardiac hypertrophy, fibrosis and dysfunction. Meanwhile, TBC1D25 overexpression in both H9C2 cells and NRCMs alleviate Angiotensin II-induced cardiomyocyte hypertrophy *in vitro*. Moreover, TBC1D25 deficiency increases the phosphorylation levels of TAK1 and its downstream molecular (JNK and p38), whereas overexpressed TBC1D25 inhibits phosphorylation of TAK1, JNK and p38. And TAK1 is the key molecule in this process. Furthermore, we demonstrated that TBC1D25 could directly interacts with TAK1 by immunoprecipitation assay and GST pull-down assay, and the interaction needs the amino acids from at least 138 to 226 in the C-terminal region of TBC1D25 and from 1 to 300 in the C-terminal region of TAK1. We conclude that TBC1D25 suppresses pathological cardiac remodeling via regulating TAK1-JNK/p38 signaling pathway, which suggests that TBC1D25 will likely become a promising therapeutic target for heart failure.

## Introduction

Heart failure (HF), a leading cause of morbidity and mortality in patients over 65 years of age, is a growing public health problem worldwide. Multiple pathological stimuli, such as hypertension, myocardial infarction or ischemia, cardiomyopathy, structural heart diseases, and other cardiovascular diseases are main risk factors in the progression of heart failure^1^, ^2^. Meanwhile, cardiac remodeling is a chronic maladaptive response to the pathological stimulus (stress or volume overload, neurohumoral factors, biomechanical stress and so on) which appears in the process of heart failure. Cardiac remodeling is characterized by structure rearrangement of heart and vessel which refers to re-expression of fetal cardiac genes (such as genes coding natriuretic peptides and the β-myosin heavy chain), progressive myocardial hypertrophy, apoptosis, interstitial fibrosis, vascular dysfunction. And multitudinous cell types and signaling cascades are involved in the process of cardiac remodeling. Prevailing studies suggest that cardiac remodeling is a major early event in heart failure 3. Although extensive studies have suggested that reversing or preventing cardiac remodeling is the key of HF therapy, existing therapeutic strategy against cardiac remodeling can't satisfy the need of clinical requirement. The efficacy of anti-cardiac remodeling is desperately needed to improve. In addition, the exact mechanisms of cardiac remodeling are not fully clarified yet^4^. Thus, elucidating the molecular mechanism of cardiac remodeling and identifying new therapeutic targets to prevent or reverse cardiac remodeling and heart failure are urgently needed.

Recently, the great progress in molecular biology techniques has made it possible to better understand molecular mechanism and identify new therapeutic targets involved in HF. TBC1Domain Family Member 25 (TBC1D25), also known as OATL1, is a protein contains a TBC (Tre-2/Bub2/Cdc16)/RAB-GTPase-activating protein (GAP) domain. It is identified as a protein participated in autophagy in previous studies 5, 6. Previous studies have shown that TBC1D25 is a LC3-banding protein, which could localize at LC3-positive isolation membranes and autophagosomes. And it plays an inhibitory role in the maturation step of autophagosomes through binding to LC3^5^-^7^. Relevant studies even found that TBC1D25 could expressed in cytoplasm of human mature Osteoclasts^5^. However, the role of TBC1D25 in cardiovascular diseases has not previously been investigated. Surprisingly, in our study, the expression of TBC1D25 was highly upregulated during pathological cardiac remodeling, which is unrecognized previously. In order to illuminate the function of TBC1D25 in cardiac remodeling, we used TBC1D25-KO and their wild-type control mice to induce pathological cardiac hypertrophy by transverse aortic constriction (TAC). And *in vitro*, TBC1D25-overexpressing and control cardiomyocytes were treated with Angiotensin II. The results indicated that TBC1D25 could negative regulate myocardial fibrosis, cardiac dysfunction and hypertrophy. Furthermore, we provided evidence that TBC1D25 could directly inhibit TAK1 phosphorylation, and protect against cardiac remodeling through TAK1- JNK/p38 signaling.

## Results

### TBC1 Domain Family Member 25 (TBC1D25) expression is upregulated in response to pressure overload

**Figure 1:**
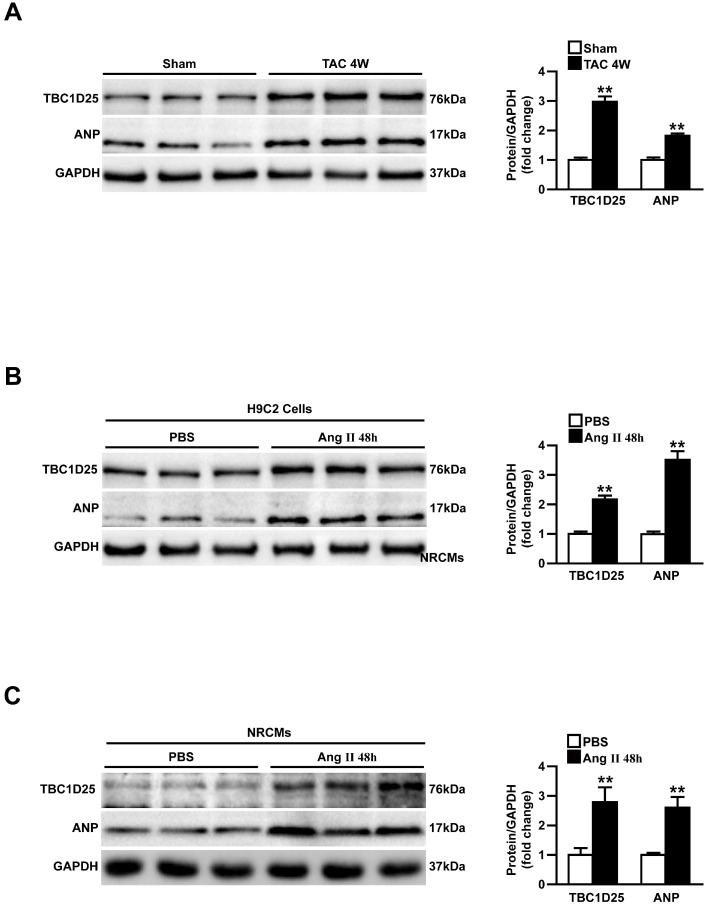
** Upregulation of TBC1D25 expression in model of cardiac hypertrophy.** (A) Western blots (left) and quantification (right) of TBC1D25 and ANP protein levels from WT mice subjected to sham surgery or TAC-induced cardiac hypertrophy at 4 weeks, n =3 mice per experimental group. Two-tailed Student's t-test. **P<0.01 vs sham. (B-C) The TBC1D25 and ANP protein levels of H9C2 cardiomyocytes or neonatal rat cardiomyocytes (NRCMs) in phosphate-buffered saline (PBS) controls, and Ang II-treated groups at 48 hours. Data are representative of at least 3 independent experiments. Two-tailed Student's t-test. **P<0.01 vs sham. All data are presented as mean ±S.D.

To investigate the potential role of TBC1D25 in cardiac hypertrophy, we first analyzed the altered expression levels of TBC1D25 in pressure overload mouse heart. In Figure 1A, the Western blot analysis demonstrated that the protein level of TBC1D25 was upregulated in hearts of mice subjected to transverse aortic constriction (TAC). Atrial natriuretic peptide (ANP), a hypertrophic marker protein was also increased dramatically in these mice (Figure 1A). Furthermore, *in vitro* study, TBC1D25 and ANP protein levels were significantly increased in H9C2 cells or neonatal rat cardiomyocytes (NRCMs) stimulated by Ang II (Figure 1B and 1C). Taken together, these findings indicated that TBC1D25 may participate in pathological cardiac remodeling.

### TBC1D25 deficiency accelerates cardiac hypertrophy induced by TAC

**Figure 2:**
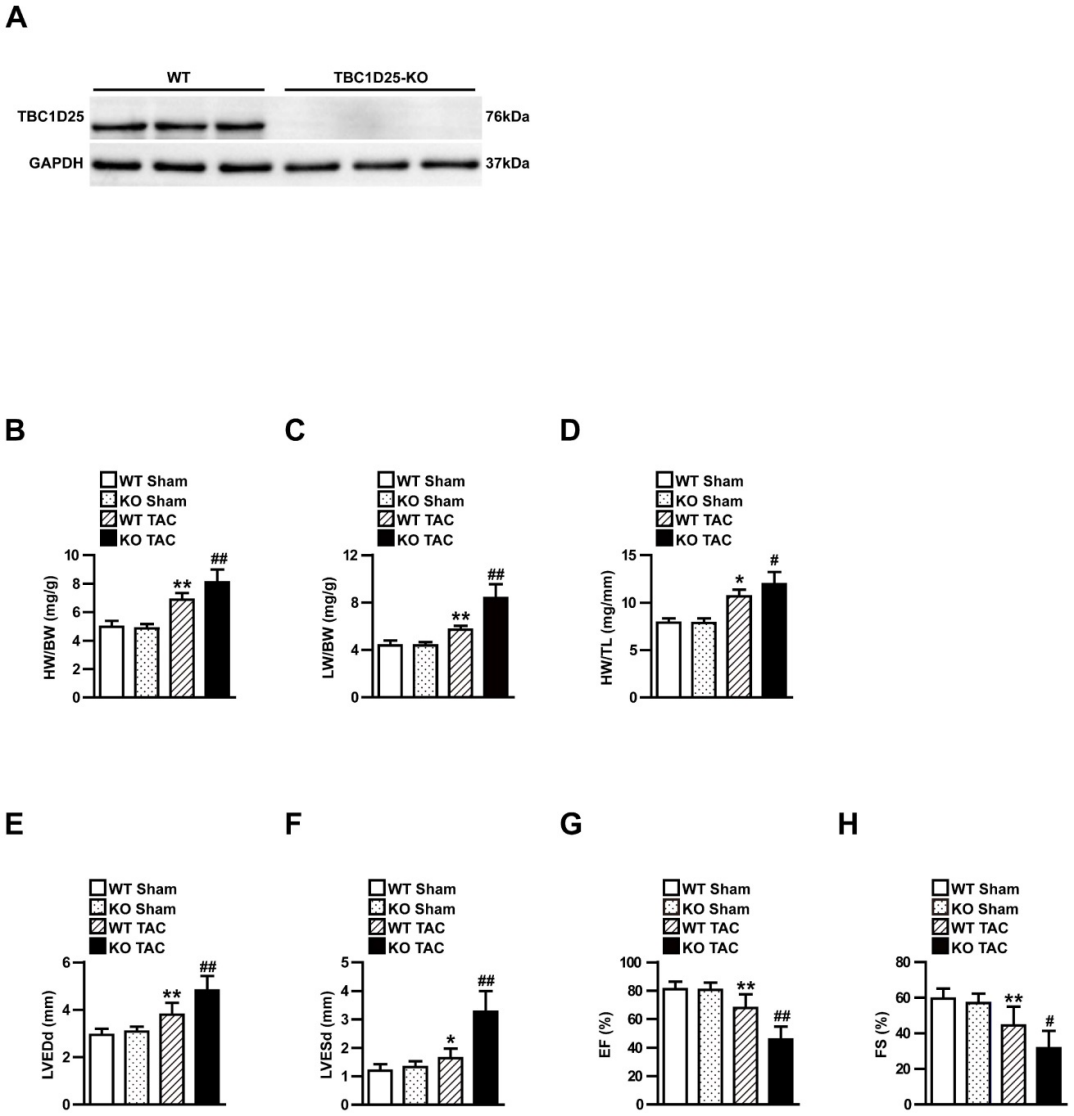
** TAC-induced cardiac hypertrophy in WT and TBC1D25-KO mice.** (A) The comparison of TBC1D25 expression in TBC1D25-KO mice and WT control mice. (B-D) The ratios of heart weight (HW)/body weight (BW), lung weight (LW)/BW, and HW/tibia length (TL) in WT and TBC1D25-KO mice at 4 weeks after sham or TAC surgery. n=11 in per groups. (E-H) Left ventricular end-diastolic diameter (LVEDd), left ventricular end-systolic diameter (LVESd), EF% and FS% were measured by echocardiography in WT and TBC1D25-KO mice subjected to sham or TAC. n=11 mice per experimental group. The data were analysed with two-tailed Student's t-test. *P<0.05 vs WT sham, **P<0.01 vs WT sham, ^#^*P*<0.05 vs WT TAC, ^##^*P*<0.01 vs WT TAC. All data are presented as mean ±S.D.

TBC1D25 expression was significantly increased in experimental cardiac hypertrophy, which indicated TBC1D25 may regulate pathological cardiac remodeling in this process. To further evaluate the function of TBC1D25 in cardiac remodeling, TBC1D25 -KO mice were utilized in our research. Compared with wild-type (WT) control mice, the loss of TBC1D25 protein in TBC1D25-KO mice was confirmed by Western blotting (Figure 2A). Subsequently, TBC1D25-KO mice and WT mice were subjected to TAC or sham operation. The heart structure and function were examined after 4 weeks of TAC or sham operation. No comparable differences were noticed in the sham-operated TBC1D25-KO mice and WT controls. Compared with WT mice, TBC1D25-KO mice manifested increased ratios of heart weight to body weight (HW/BW; Figure 2B), lung weight to body weight (LW/BW; Figure 2C) and heart weight to tibia length (HW/TL; Figure 2D). Furthermore, the echocardiography assay confirmed that left ventricular end-diastolic diameter (LVEDd), left ventricular end-systolic diameter (LVESd), EF% and FS% were significantly decreased in TBC1D25-KO mice compared with WT control mice (Figure 2E-H).

**Figure 3:**
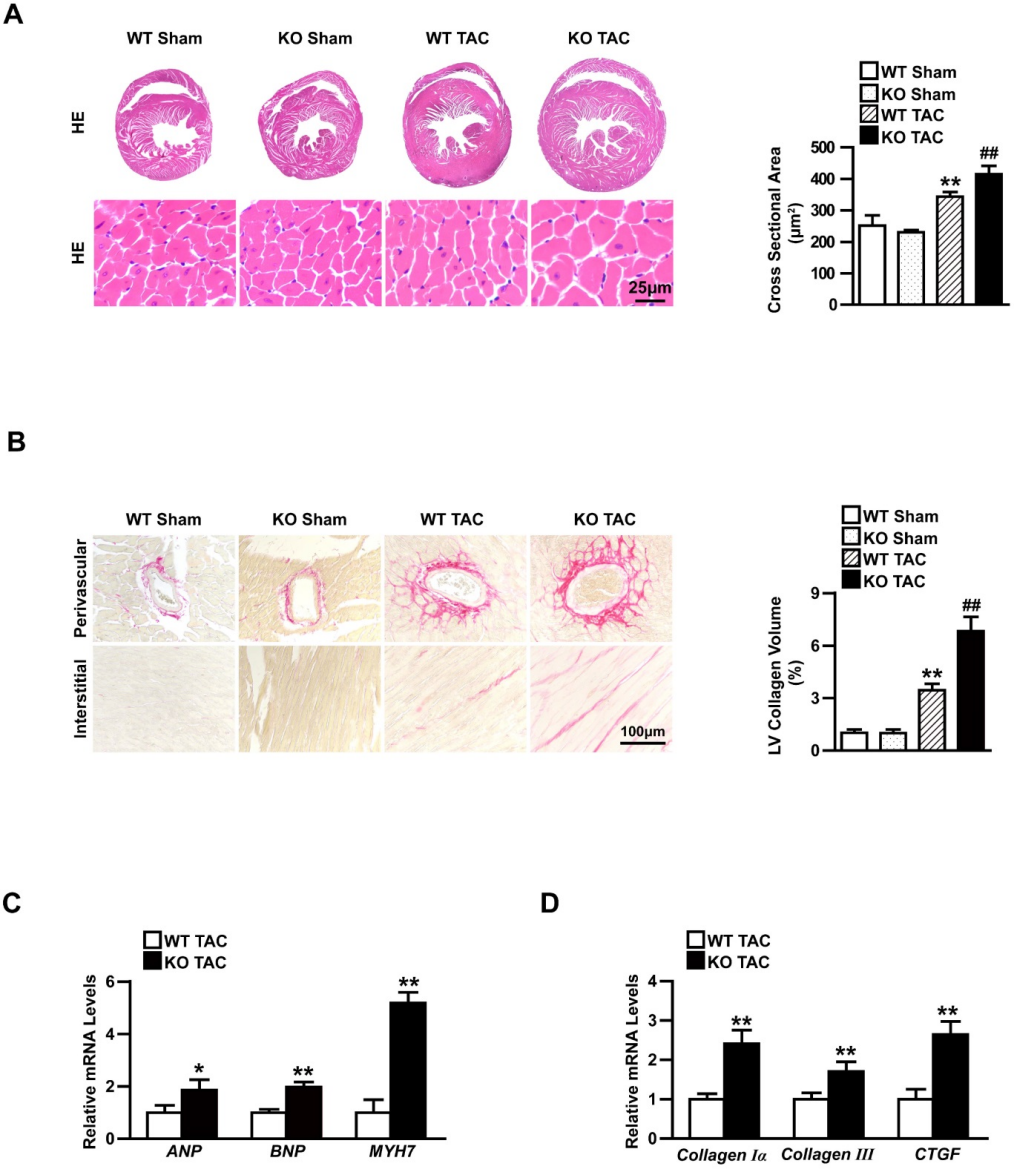
** Knockout TBC1D25 exacerbates cardiac hypertrophy induced by increasing afterload in left ventricle.** (A) Hematoxylin and eosin staining (left) and quantification (right) of WT and TBC1D25-KO mice hearts after 4 weeks of sham or TAC. Left, n=6 mice per experimental group. Right, n=100-200 cells per experimental group. Scale bars 50 μm. (B) Picrosirius red (PSR) stained perivascular and interstitial area (left) and LV collagen volume (right) in each group. Left, n=6mice per experimental group. Right, n=50 fields per experimental group. Scale bar, 100 μm. **P<0.01 vs WT sham, ^##^*P*<0.01 vs WT TAC. (C-D) The mRNA levels of hypertrophic markers (ANP, BNP, and MYH7) and fibrotic markers (Collagen Iα, Collagen III and CTGF) in the hearts of WT and TBC1D25-KO mice after TAC treatment. n=3-4 mice per experimental group. Two-tailed Student's t-test. *P<0.05 vs WT group, **P<0.01 vs WT group. All data are presented as mean ±S.D.

Moreover, the heart cross-sections of WT and TBC1D25-KO mice were stained with haematoxylin and eosin (H&E) or picrosirius red (PSR). After 4 weeks of TAC, TBC1D25-KO mice exhibited a significantly larger heart and cardiomyocytes cross-sectional area (Figure 3A) and more prominent interstitial and perivascular fibrosis(Figure 3B). While, there were no comparable differences in the sham-operated TBC1D25-KO mice and WT mice (Figure 3A-B). RNA expression of hypertrophy markers (ANP, BNP and MYH7) and fibrosis markers (Collagen Iα, Collagen III and CTGF). The TBC1D25-KO mice showed greatly increased mRNA expression levels of these markers compared with WT mice (Figure 3C and 3D). These data demonstrated that loss of TBC1D25 exacerbated cardiac dilation and dysfunction greatly.

### TBC1D25 suppresses angiotensin II-induced cardiomyocytes hypertrophy *in vitro*

**Figure 4:**
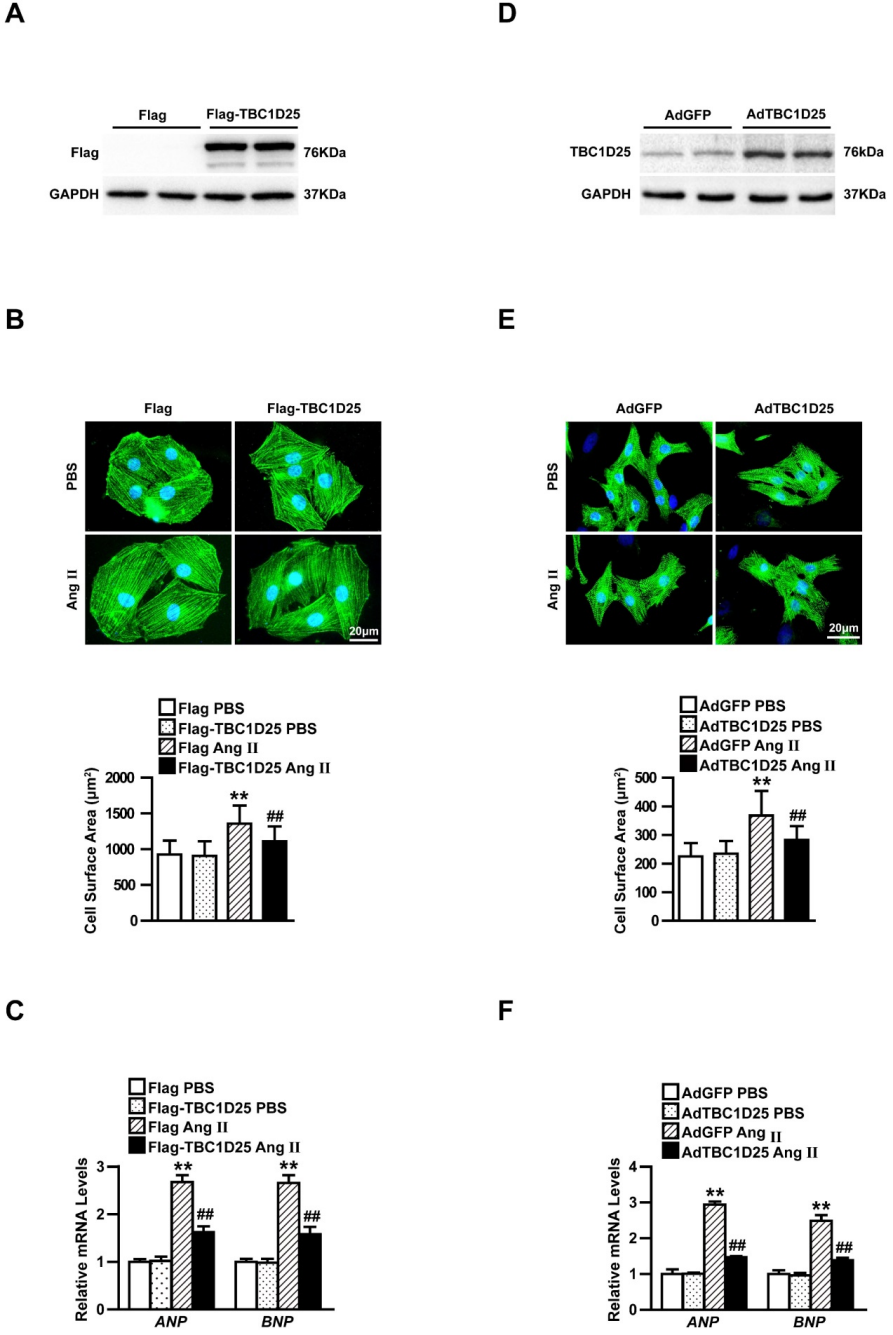
** TBC1D25 affects Ang II-induced cardiomyocyte hypertrophy *in vitro*.** (A) Immunoblotting of Flag-TBC1D25 in H9C2 cardiomyocytes. n = 2 samples per group. (B) Representative images of α-actinin (green) and DAPI (blue) stained H9C2 cells in each group treated with PBS or Ang II (above). Quantification of cell surface area (below). Above, n=3 independent experiments. Below, n=50 cells per group. Scale bar, 20 μm. (C) Relative mRNA expression of ANP and BNP in each group treated with Ang II or PBS. n=3 independent experiments. One-way ANOVA. **P<0.01 vs Flag PBS group, ^##^*P*<0.01 vs Flag Ang II group. (D) NRCMs infected with AdTBC1D25 and control (AdGFP) were analyzed by Western blotting. (E) Representative images of NRCMs infected with AdTBC1D25 or AdGFP after treatment with Ang II or PBS (above). Quantification of cell surface area (below). Above, n=3 independent experiments. Below, n=50 cells per group. Scale bar, 20 μm. (F) ANP and BNP mRNA levels in NRCMs treated Ang II or PBS. n=3 independent experiments. One-way ANOVA. **P<0.01 vs AdGFP PBS group, ^##^P<0.01 vs AdGFP Ang II group. All data are presented as mean ±S.D.

Cardiomyocyte enlargement is the mainly characteristic changes of cardiac remodeling^8^. We next examined the contribution of TBC1D25 to cardiac hypertrophy in cardiomyocytes. To address this issue, H9C2 cells and neonatal rat cardiomyocytes (NRCMs) were investigated. H9C2 cells have similar biological characteristics (including morphology, protein expression, signaling and hypertrophy related characters) with adult cardiomyocytes^9^. Many studies use H9C2 cells to investigate cardiac hypertrophy^10^, ^11^. In this study, we infected viruses containing TBC1D25 and control particles with H9C2 cardiomyocytes to overexpress TBC1D25 (Figure 4A). The infected cells were selected and then treated with Ang II or PBS for 48 h. Then, the cell surface area and expression of ANP, BNP were detected. The data demonstrate that Ang II had pro-hypertrophic effect *in vitro*. However, TBC1D25 overexpression dramatically decreased the hypertrophic phenotypes induced by Ang II, compared with control cells (Figure 4B and 4C). To further confirm the function of TBC1D25 in cardiomyocyte hypertrophy, NRCMs were isolated and infected with AdTBC1D25 or AdGFP, respectively (Figure 4D). Similarly, AdTBC1D25-infected NRCMs exhibited decreased individual cell size and mRNA levels of hypertrophic markers (Figure 4E-F). These findings confirmed that TBC1D25 plays an important antihypertrophic role at the cellular level.

### TBC1D25 regulates TAK1 signaling during cardiac hypertrophy both *in vivo* and *vitro*

**Figure 5:**
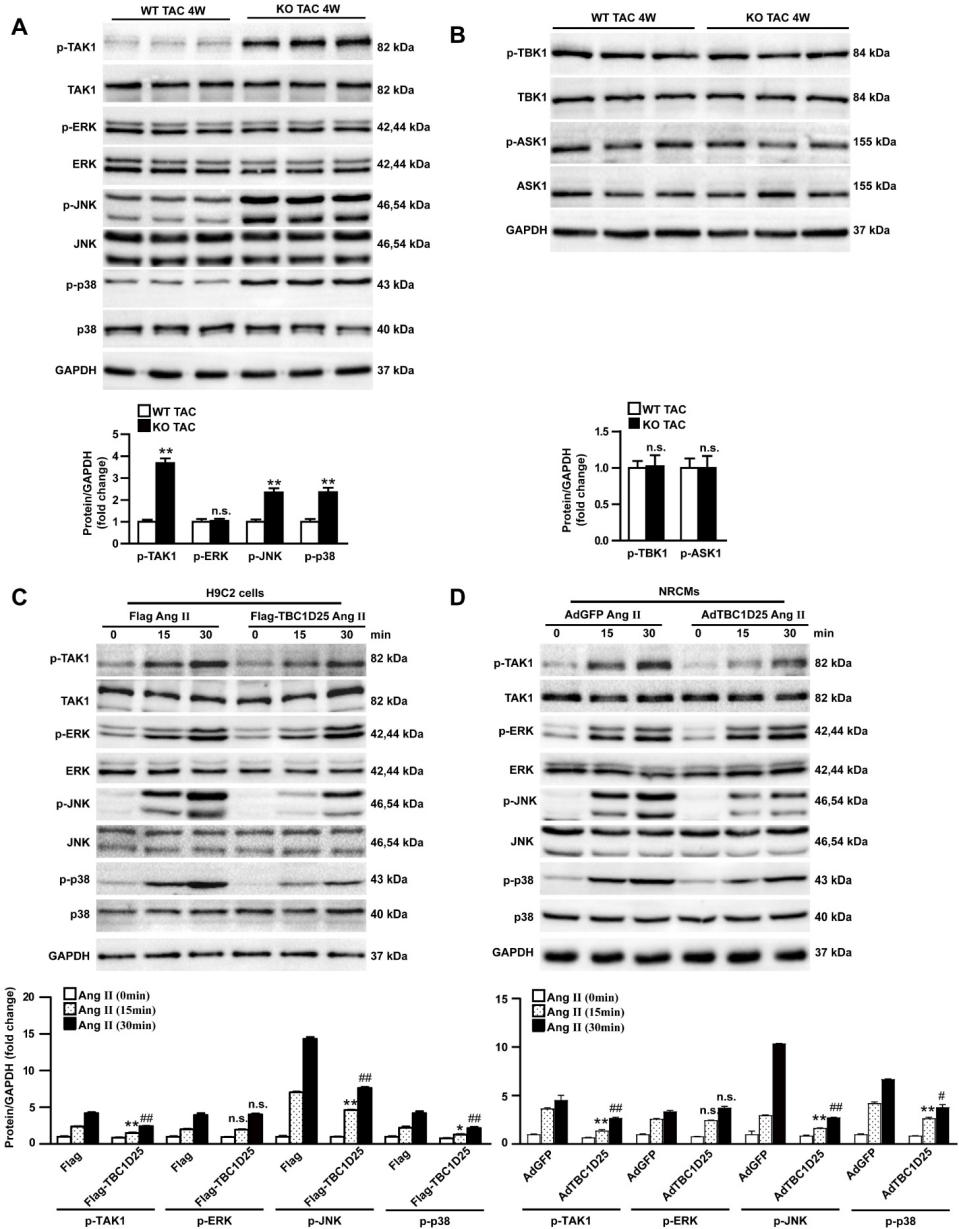
** TBC1D25 inhibits TAK1signalling pathway.** (A) Representative western blots (top) and quantification (bottom) of phosphorylated and total TAK1, ERK, JNK and p38 in WT and TBC1D25-KO mice subjected to TAC. n=3 mice per experimental group. (B) Representative western blots (top) and quantification (bottom) of phosphorylated and total TBK1 and ASK1 in WT and TBC1D25-KO mice subjected to TAC. n= 3 mice per experimental group. Two-tailed Student's t-test. **P<0.01 vs WT group. n.s. indicates no significance. (C) Representative western blots (top) and quantification (bottom) of phosphorylation and total TAK1, ERK, JNK and p38 in H9C2 cardiomyocytes infected with lenti-GFP or lenti-TBC1D25 and treated with Ang II for the indicated times. n=3 independent experiments. One-way ANOVA. *P<0.05 vs Flag Ang II (15min) group, **P<0.01 vs Flag Ang II (15min) group, ^##^*P*<0.01 vs Flag Ang II (30min). n.s. indicates no significance. (D) Representative western blots (top) and quantification (bottom) of phosphorylation and total TAK1, ERK, JNK and p38 in NRCMs infected with AdGFP or AdTBC1D25 and treated with Ang II for the indicated times. n=3 independent experiments. One-way ANOVA. **P<0.01 vs AdGFP Ang II (15min) group,^ #^*P*<0.05 vs AdGFP Ang II (30min) ^##^*P*<0.01 vs AdGFP Ang II (30min). n.s. indicates no significance. All data are presented as mean ±S.D.

The protective role of TBC1D25 in cardiac hypertrophy had been demonstrated. Furthermore, we explored the molecular mechanisms by which TBC1D25 regulated cardiac hypertrophy. Many researches proved that MAPK signaling pathways played an important role in cardiac remodeling^12^, ^13^. So we evaluated the potential involvement of MAPK pathways in this process. Therefore, we monitored the expression of TAK1, ERK, JNK, and p38 in heart of TAC-operated WT and TBC1D25-KO mice. Our data indicated that the phosphorylation levels of TAK1, JNK, and p38 were significantly increased in TBC1D25-KO mice, compared with TAC-operated WT mice. But phosphorylated ERK was not affected by TBC1D25 deficiency (Figure 5A). At the same time, we detected phosphorylation and total level of TBK1 and ASK1 *in vivo* study. And the results demonstrated that TBC1D25 knockout did not affect phosphorylation of TBK1 and ASK1(Figure 5B). *In vitro* experiments, TBC1D25-overexpressing H9C2 cells and NRCMs were treated with Ang II at specified points in time. Our data indicated that TBC1D25 overexpression remarkably suppressed the level of phosphorylated TAK1, JNK, and p38 (Figure 5C-D).

### Inhibiting TAK1 is the key step of cardiac remodeling regulated by TBC1D25

**Figure 6:**
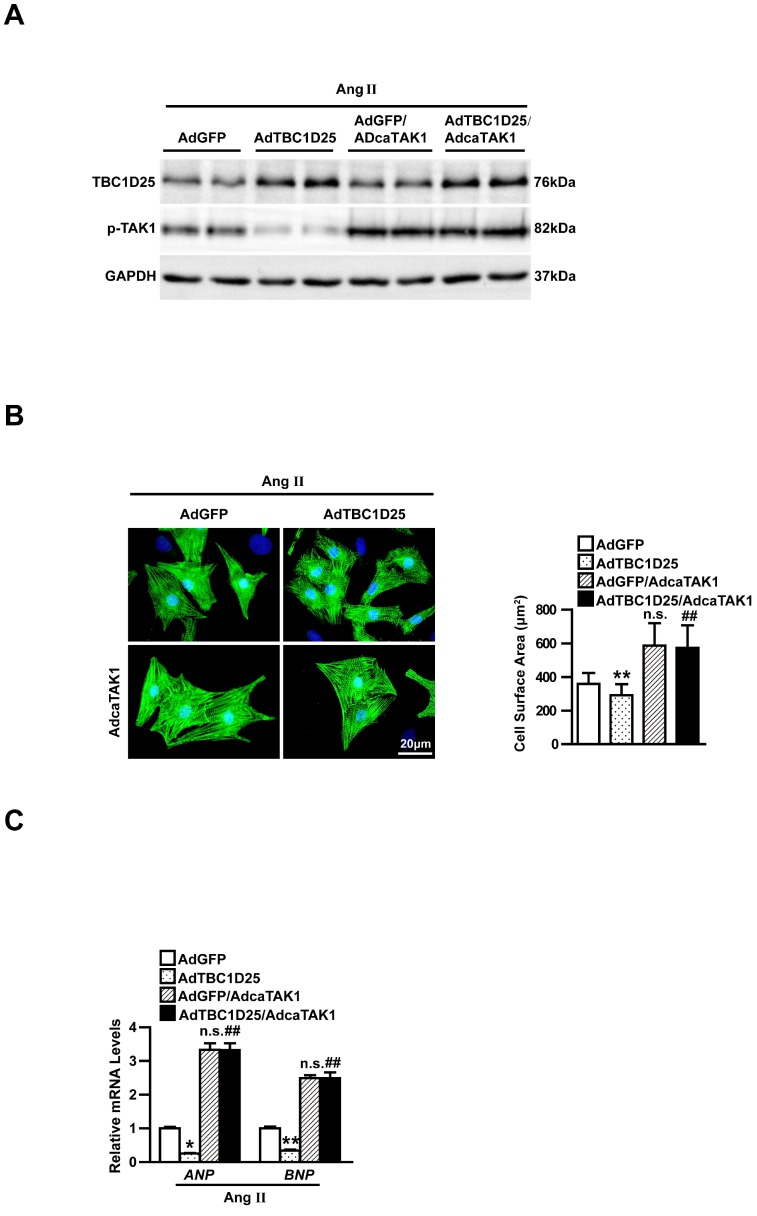
** Activated TAK1 reverses the protective effects of TBC1D25 on cardiomyocytes hypertrophy induced by Ang II.** (A) Representative western blots of TBC1D25 and in AdGFP, AdTBC1D25, AdGFP+AdcaTAK1 or AdTBC1D25+AdcaTAK1 infected NRCMs. (B) Representative images of cardiomyocytes (immunostained with the α-actinin antibody) infected with AdGFP, AdTBC1D25, AdGFP+AdcaTAK1 or AdTBC1D25+AdcaTAK1 after treatment with Ang II (left). Quantification of cell surface area (right). Left, n=3 independent experiments. Right, n=50 cells per group. Scale bar, 20 μm. (C) ANP and BNP mRNA levels in NRCMs treated Ang II in each group. *P<0.05 vs AdGFP group, **P<0.01 vs AdGFP group, ^##^*P*<0.01 vs AdTBC1D25 group. n.s. indicates no significance.

Furthermore, to verify whether inhibition of TAK1 is required in TBC1D25 mediated anti-hypertrophic response, we infected AdcaTAK1 (adenovirus expressing constitutively active TAK1) into AdGFP or AdTBC1D25 infected NRCMs, respectively (Figure 6A). AdcaTAK1 infection could partially reverse the protective effects of AdTBC1D25 in Ang II-induced cardiomyocyte hypertrophy (Figure 6B-C). These findings robustly verified that TBC1D25 regulate pathological cardiac remodeling through TAK1 signaling pathway.

**Figure 7:**
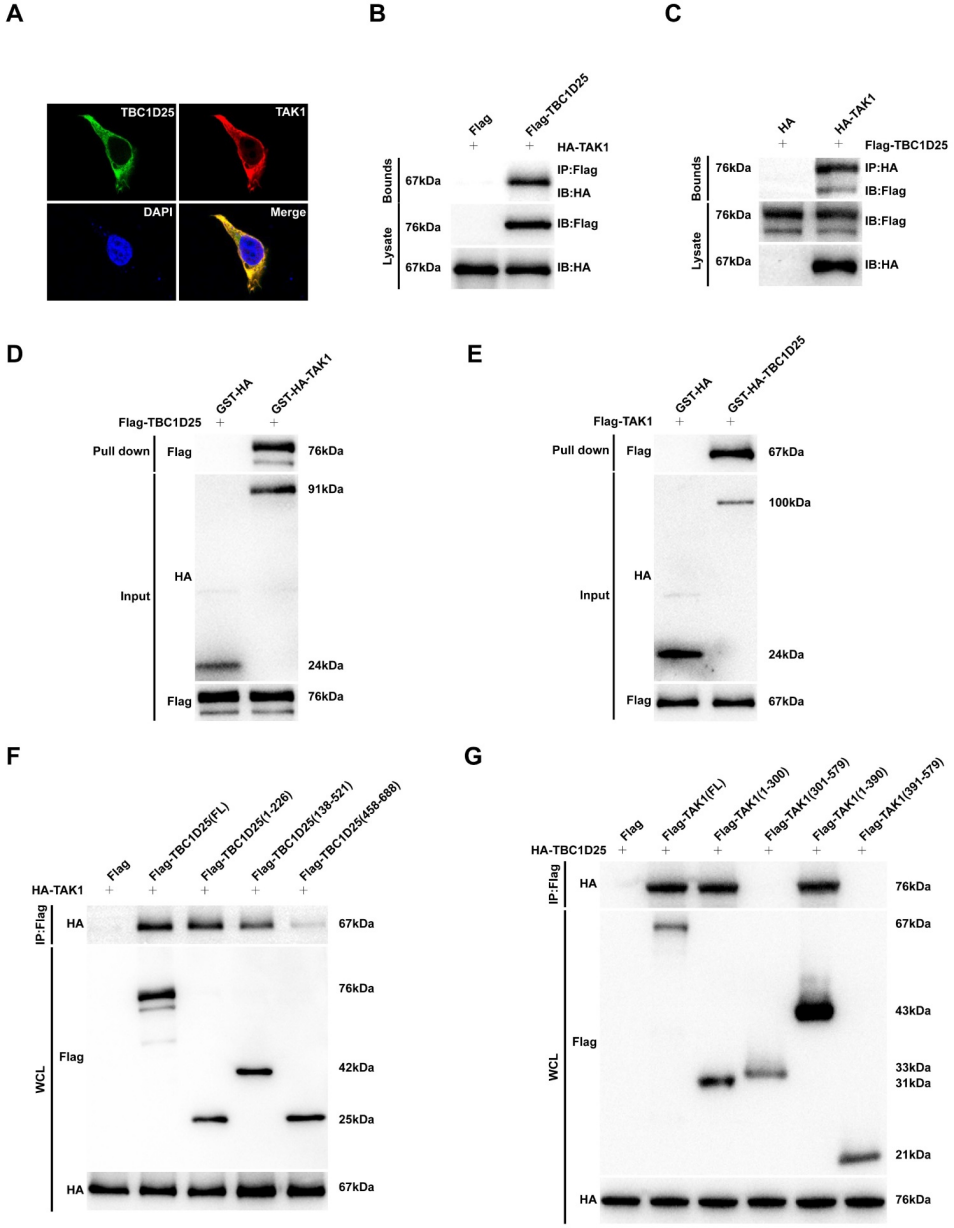
** The direct interaction between TBC1D25 and TAK1.** (A) The co-localization of TBC1D25 and TAK1 in 293T cells. The staining of TBC1D25 is green, TAK1 is red and DAPI is blue in representative confocal images. (B-C) The TBC1D25 and TAK1 interaction was detected by Immunoprecipitation (IP) assays. 293T cells were co-transfected with Flag-TBC1D25 and HA-TAK1. 48 hours later, harvested cell lysates were subjected to IP with antibodies against Flag (B) or HA (C). (D-E) The direct interaction between TBC1D25 and TAK1 were verified by GST pull-down assays. GST-tagged proteins were incubated with immunopurified Flag-tagged proteins and then immunoblotted. (F-G) Co-IP of the full-length and truncated mutants of TBC1D25 (F) or TAK1 (G) using a HA or Flag antibody, respectively.

**Figure 8:**
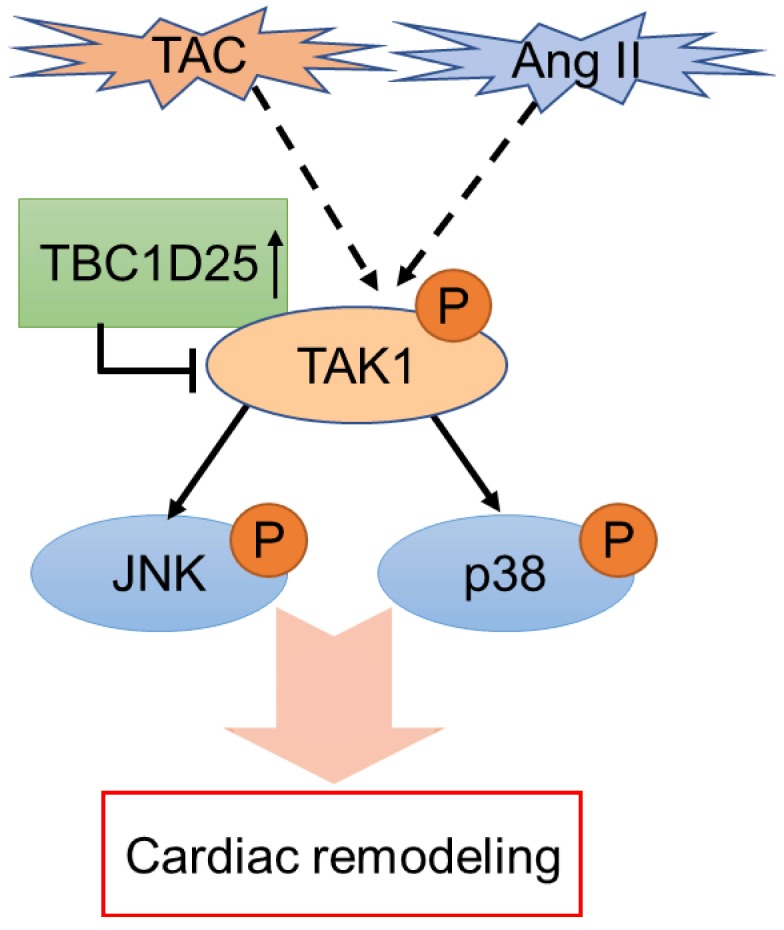
** The schematic diagram of TBC1D25 regulates cardiac remodeling.** TBC1D25 regulates cardiac remodeling by inhibiting TAK1-JNK/p38 signaling pathway. TAC, transverse aortic constriction; Ang II, Angiotensin II.

To further investigate the molecular mechanism of TBC1D25-regulated cardiac remodeling. We examined whether TBC1D25 could directly regulates TAK1 activation. The result showed that TBC1D25 and TAK1 were completely co-localized in the cytoplasm of 293T cells by immunofluorescent staining (Figure 7A). And then, we transfected 293T cells with Flag-tagged TBC1D25 and HA-tagged TAK1. The direct interaction of TBC1D25 and TAK1 was demonstrated by Immunoprecipitation assay (Figure 7B-C). Furthermore, Glutathione S-transferase (GST) pull-down assays were performed to confirm this interaction between TBC1D25 and TAK1(Figure 7D-E). Subsequently, we constructed a series of TBC1D25 and TAK1 mutants to further identify the binding domain for TBC1D25 and TAK1 direct interaction. The results showed that the amino acids from at least 138 to 226 in the C-terminal region of TBC1D25 and from 1 to 300 in the C-terminal region of TAK1 was required for TBC1D25 and TAK1 direct interaction(Figure 7F and 7G). All these data indicated that TBC1D25 directly regulate TAK1-JNK/p38 signaling pathway, which is important in the process of cardiac remodeling (Figure 8).

## Discussion

Heart failure is one of the most common cardiovascular diseases with extremely high morbidity and mortality. Mainstream researches suggest that cardiac remodeling, the major early event of heart failure, is the key target in HF therapy^14^. The identification of molecular targets for reversing or preventing cardiac remodeling is necessitated in HF treatments.

Here, we demonstrate the important function of TBC1D25 in pathological cardiac remodeling, which is unrecognized previously. TBC1D25 (also known as OATL1), mainly localized outside autophagosomes, is a protein contains a TBC (Tre-2/Bub2/Cdc16) /RAB-GTPase-activating protein (GAP) domain. TBC1D25 is identified as a LC3-banding protein, which could localize at LC3-positive isolation membranes and autophagosomes. Previous researches have shown that TBC1D25 plays an inhibitory role in the maturation step of autophagosomes through binding to LC3 5-7. And Klinck et al confirm that TBC1D25 could expressed in cytoplasm of human mature Osteoclasts^5^. However, our present study showed that TBC1D25 is upregulated during the development cardiac hypertrophy and plays a very important role in this process. TBC1D25 knockout in mice leads to more severe myocardial fibrosis, cardiac dysfunction and hypertrophy. Moreover, overexpression of TBC1D25 could suppress Ang II-induced cardiomyocyte hypertrophy *in vitro*. These findings indicated a new function of TBC1D25 in cardiac remodeling, which is undiscovered in previous study. Furthermore, our results indicate that TAK1 is the key molecule in this process. TBC1D25 could directly interact with TAK1 and significantly inhibit TAK1 phosphorylation in the development of cardiac hypertrophy. JNK and p38, the downstream molecular cascades of TAK1^15^, is suppressed by TBC1D25 through regulation of TAK1. Meaningfully, it seems highly probable that the anti-cardiac hypertrophic effect of TBC1D25 is associated with suppression of TAK1-JNK/p38 signaling pathway. Meanwhile, to determine whether other proteins participate in cardiac remodeling regulated by TBC1D25. we detected expression of phosphorylated and total TBK1 and ASK1. TBK1 and ASK1. TBK1 belongs to IκB kinase (IKK) family which is ubiquitously expressed in multiple tissues^16^. Previous studies have shown that TBK1 participate in development of cardiac hypertrophy through AKT signaling pathway^17^, ^18^. Apoptosis signal regulating kinase 1 (ASK1) is considered as a participant of cardiac remodeling in many studies 19, 20. It is a member of mitogen-activated protein kinase kinase kinase (MAPKKK) family, which regulates cell death through JNK, p38 and other effectors^21^, ^22^. However, in our study, the activation of TBK1 and ASK1 were not affected by TBC1D25, which means that TBK1 and ASK1 did not participate in the process of cardiac remodeling regulated by TBC1D25. That means TBC1D25 regulate cardiac remodeling primary through TAK1-JNK/p38 signaling pathway.

Transforming growth factor-β-activated kinase 1 (TAK1), also referred to as mitogen-activated protein kinase kinase kinase 7 (MAP3K7), controls a variety of cell functions including transcription regulation, inflammation, and apoptosis^23^-^25^. Dou Zhang et al demonstrated the role of TAK1 in heart failure. They found that TAK1 was upregulated after aortic banding and promoted interstitial fibrosis, myocardial dysfunction and cardiac hypertrophy^26^. Other researches proved that cardiac-specific overexpression of TAK1 exaggerates pressure overload induced cardiac hypertrophy and dysfunction, indicating that TAK1 is a pivotal regulator of cardiac hypertrophy^24^, ^26^. Meanwhile, previous studies found that TAK1 was regulated by TRAF6, TNF-α, TRAIL, IL-1and LPSs^15^, ^27^. And Yan-Xiao Ji et al found that TRAF6 could exacerbate cardiac hypertrophy via TAK1-dependent signaling pathway^26^. In their study, TRAF6 regulated pathological cardiac hypertrophy dependent on directly interacting with TAK1 and promoting TAK1 ubiquitination. Significantly, our study demonstrated for the first time that TBC1D25 inhibits cardiac remodeling though interacting with TAK1 directly. Besides, multiple studies strongly indicated that JNK and p38 were important regulators of cardiac remodeling, which promoted myocardial hypertrophy and dysfunction^13^, ^28^, ^29^. JNK and p38 are mitogen-activated protein kinases (MAPKs) family members, which participate in various pathological and physiological processes^30^. In models of cardiac hypertrophy, activated TAK1-JNK/p38 signaling pathway aggravated functional and structural cardiac remodeling^26^. We also observed that phosphorylation levels of JNK and p38 were correspondingly changed when the activation of TAK1 was altered. JNK and p38 participated in cardiac hypertrophy and disfunction both *in vivo* and *in vitro* studies^31^. Collectively, TBC1D25 negative regulate cardiac remodeling via inhibiting TAK1-JNK/p38 signaling pathway. In addition, ERK is another MAPKs family member, which is regulated by TAK1^32^. Many researches indicated that ERK plays a promoting role in cardiac hypertrophy process^33^, ^34^. But in our study, we did not observe changes in ERK activity both *in vivo* and *in vitro*. This means that TBC1D25, even TAK1 did not affect the activation of ERK in this study, which had not been understood and needed further research. Additionally, the molecular mechanism of cardiac remodeling is extremely complicated, future studies are needed to elucidate the upstream of TBC1D25 in this process and clarify whether TBC1D25 participate in the development of other cardiovascular diseases.

## Conclusions

In summary, our *in vivo* and *in vitro* experiments demonstrated that TBC1D25 protects against pathological cardiac remodeling. Knockout of TBC1D25 aggravates interstitial fibrosis, myocardial dysfunction and cardiac hypertrophy. Conversely, overexpression of TBC1D25 mitigates cardiac remodeling. The cardioprotective effect of TBC1D25 involves the suppression of TAK1-JNK/p38 signaling pathway. In brief, TBC1D25 will probably become a new therapeutic or research target in cardiac remodeling.

## Materials and Methods

### Animals use for study

Experimental procedures were performed according to the NIH Guide for the Care and Use of Laboratory Animals published by the US National Institute of Health (NIH publication, 8th edition, 2011). All animal usage protocols were approved by the Animal Care and Use Committee of The First Affiliated Hospital of Zhengzhou University. The related procedures were conducted in accordance with the National Institutes of Health Guide for the Care and Use of Laboratory Animals. Constitutive TBC1D25 knock-out (TBC1D25-KO) mice were purchased from the Texas A&M Institute for Genomic Medicine.

### Animal surgery

Cardiac hypertrophy was induced in mice through partial transverse aortic constriction (TAC) of aortic arch, as previously described with some adaptations^35^. Briefly, in both groups, 9-to 11-week-old male mice were fixed in a supine position after anesthetized with sodium pentobarbital via an intraperitoneal injection, and the skin in the middle of the chest was opened to expose the aortic arch through the right side of clavicle after toe pinch reflex disappeared. Body temperature was maintained as close as possible to 37.0 °C throughout the experiment using a self-regulating heating pad. Subsequently, a specific needle (26-G for body weights of 25-27 g) was placed on the aortic arch and ligating with 7-0 silk suture at same degree, then needle was removed rapidly before the closure of the skin. Mice were observed until recovery in a 37.0 °C heated cage.

### Echocardiographic assessment

Mice were anesthetized with isoflurane (1.5-2%), and echocardiography was performed using a MyLab 30CV ultrasound system (Biosound Esaote Inc.) using a 15-MHz transducer. The size of the left ventricular (LV) cavity and LV wall thickness were acquired from at least 3 consecutive cardiac cycles. The end-systole and end-diastole were defined as the phases in which the smallest or largest LV area was obtained, respectively. The LV end-diastolic dimension (LVEDd) and LV end-systolic dimension (LVESd) were measured from the LV M-mode tracing, with a sweep of 50 mm/s at the mid-papillary muscle level. Fractional shortening (FS%) was calculated using the formula: FS%= (LVEDd-LVESd)/LVEDd×100%.

### Histological analysis

Hearts were obtained from experimental animals 4 weeks after TAC surgery that had been perfused with a 10% potassium chloride solution to induce cardiac arrest at the end of diastole and then harvested and fixed with a 10% formalin solution. After being embedded in paraffin, the hearts were cut into 5-μm transverse sections. Sections were stained with hematoxylin and eosin (H&E) to measure the myocyte cross-sectional area, and the abundance of collagen was assessed after Picrosirius red (PSR) staining. Fibrosis was expressed as a percentage of the average positively stained area relative to the total area. More than 40 fields per group were examined. A quantitative digital image analysis system (Image-Pro Plus 6.0) was used for the image measurements.

### Plasmids

TBC1 Domain Family Member 25 (TBC1D25, NM_002536.4) and TAK1 (NM_003188.3) gene were amplified by nested PCR and then cloned into pHAGE-3×flag vector, or pcDNA5-flag and pcDNA5- HA vector, or GST-HA vector. Primer sequences used for cloning the coding sequences of TBC1D25 and TAK1 were as following: TBC1D25 forward, '-TCGGGTTTAAACGGATCCGGGATGGCAACAGCCTCC-3'; TBC1D25 reverse, 5'-GGGCCCTCTAGACTCGAGTCAAGATGCGGCTGTGGCCT-3'. TAK1 forward, 5'-TCGGGTTTAAACGGATCCATGTCTACAGCCTCTGCCGCCT-3'; TAK1 reverse, 5'-GGGCCCTCTAGACTCGAGTCATGAAGTGCCTTGTCGTTTCT-3'.

### Cell cultures and lentivirual infections

Primary neonatal rat cardiomyocytes (NRCMs) were prepared as described previously^36^. NRCMs isolated from the hearts of 1- to 2-day old Sprague-Dawley rats were enriched, and seeded into 6-well plates at a density of 3×10^5^ cells/well. Cultured them in DMEM/F12 containing 20 % FCS, BrdU (0.1 mM, inhibits the proliferation of fibroblasts), and penicillin/streptomycin at 37℃ for 48 h. Then, replaced the culture medium with serum-free DMEM/F12 for 12 hours. For further experiments, AdGFP (used as the non-targeting control) or AdTBC1D25 adenoviruses were generated and infected into NRCMs at a multiplicity of infection (MOI) of 10 for 24h. Similarity, AdcaTAK1 (adenovirus expressing constitutively active TAK1) were co-infected into AdGFP or AdTBC1D25 infected NRCMs respectively.

H9C2 cardiomyocytes and HEK-293T cell lines were maintained in DMEM medium containing 10% fetal bovine serum and 1% pencillin/streptomycin. For lentiviral infection, recombinant constructs were firstly co-transfected into HEK-293T cells with lentiviral helper plasmids (pMD2.G and psPAX2) and PEI transfection reagent. After 6 to 16 hours, fresh media were replaced and lentiviral particles were harvested 48 hours later and then used for target cell infection. Briefly, viruses containing TBC1D25 and control particles were added to H9C2 cardiomyocytes in the presence of polybrene (8μg/mL) respectively. Infected cells were selected by puromycin (2μg/mL) for 48 hours and then used for indicated assays.

### Immunofluorescence staining

Immunofluorescence staining was performed to determine the surface area of the cell. H9C2s and NRCMs were stimulated with PBS or Ang II (1 μmol/L) for 48 h under a condition of 37.0 °C, 5% CO_2_ and fixed with 3.7% formaldehyde. After permeabilization with 0.1% Triton X-100 in PBS and blocking with a 10% BSA solution at room temperature, cells were immunostained with an α-actinin antibody (05-384, Merck Millipore, 1:100 dilution), followed by staining with a fluorescent secondary antibody (donkey anti-mouse IgG [H+L] secondary antibody, A21202, Invitrogen, 1:200). Image-Pro Plus 6.0 software was used to measure the surface area of the cell.

### Quantitative real-time (RT) PCR and Western blotting

For the RT-PCR assay, total mRNA was extracted from ventricular tissues or cells with TRIzol reagent (15596-026, Invitrogen). Then, cDNAs were reverse transcribed from RNAs using the Transcriptor First Strand cDNA Synthesis Kit (04896866001, Roche). Quantitative real-time PCR was used to detect the expression of selected genes with SYBR Green PCR Master Mix (04887352001, Roche). Glyceraldehyde-3-phosphate dehydrogenase (GAPDH) was used as the reference gene. The primer pairs used in this study are listed in Supplementary table 1.

For the Western blot analyses, total proteins were extracted from ventricular tissues or cell samples using RIPA lysis buffer (720 μL of RIPA buffer, 20 μL of phenylmethylsulfonyl fluoride, 100 μL of complete protease inhibitor cocktail, 100 μL of Phos-stop, 50 μL of NaF and 10 μL of Na3 VO4 in a final volume of 1 mL), and the protein concentration was determined with a BCA Protein Assay Kit (23225, Thermo Scientific). After fractionation by using SDS-PAGE, proteins were transferred to PVDF membranes and blocked with 5% non-fat milk at room temperature for 1h. After incubated with multiple antibodies overnight at 4 °C, the secondary antibodies were added next day, and bands were visualized using a Bio-Rad ChemiDoc XRS+ system (Bio-Rad). The levels of specific proteins were normalized to the levels of GAPDH on the same PVDF membrane. The antibodies used in this study are listed in Supplementary table 2.

### Immunoprecipitation (IP) assay

IP was performed as previously described^26^. Briefly, after transient transfection with plasmid, HEK-293T cells were lysed in ice-cold IP buffer (20 mM Tris-HCl, pH7.4, 150 mM NaCl, 1 mM EDTA, 1% Triton X-100) containing protease inhibitor cocktail tablets (04693132001, Roche). The cell lysates were incubated with the indicated antibody-conjugated beads (Anti-Flag M2 Magnetic Beads, M8823, Sigma; Pierce Anti-HA Magnetic Beads, 88836, Thermo Fisher Scientific) at 4°C overnight. The immunoprecipitates were subjected to immunoblotting using the indicated primary and corresponding secondary antibodies.

### GST pull-down assay

In glutathione S-transferase (GST) pull-down assay^37^, immunopurified Flag- TBC1D25 or Flag- TAK1 was incubated in elution buffer for 2 h at 4ºC. The elution buffer was composed of 50mM HEPES (pH 7.4), 500mM NaCl, 1% Triton X-100 and 3 μg μl-1 FLAG peptide (F4799, Sigma).Transfect vectors pGEX-4T-1- GST- HA- TAK1 or GST- HA- TBC1D25 into Rosetta (DE3) *Escherichia coli*, and then induced them with 1mM isopropyl-β-D-thiogalactopyranoside (IPTG) at an optical density of 600nm (OD600) of 0.6. *Escherichia coli* extracts were dealt with PBS containing protease inhibitor cocktail tablets (04693132001, Roche). Subsequently, incubated them with glutathione-sepharose 4B beads (17075601, GE Healthcare Biosciences AB) for 1h at 4 ℃. The proteins binding to the beads were washed five times with 1ml of PBS, and then incubated them for an additional 4h at 4℃ with immunopurified Flag- TBC1D25 or Flag-TAK1. Then washed the glutathione-sepharose 4B beads with 1ml of the NaCl wash buffer for three times. And the eluted proteins were analyzed by Western blotting using indicated antibodies. In the same conditions, the GST tag was used as the negative control.

Immunofluorence and confocal microscopy assays were detected as previously described 18, HEK-293T cells were cultured on gelatin-coated coverslips in 24-well plates and then co-transfected with Flag-TBC1D25 and HA-TAK1. 24h later, cells on coverslips were fixed with 4% fresh paraformaldehyde for 15 min and permeabilized subsequently with 0.2% Triton X-100 in PBS for additional 5min. Then coverslips were incubated with indicated primary and fluprescent secondary antiboies sequently. The coverslips were washed three times with PBS and stained with DAPI (10mg/ml) before mounted by mounting solution (D2522, Sigma). Fluorescence photographs were acquired and analyzed by Laser Scanning Confocal Microscopy (LSM880, ZEISS).

### Statistical analysis

Data are presented as means ± S.D. Comparisons between two groups were performed using a two-tailed Student's *t*-test. Differences among more than 2 groups were assessed using one-way analysis of variance (ANOVA), followed by the Bonferroni test (equal variances assumed) or Tamhane's T2 (equal variances not assumed) test. A value of P<0.05 was considered to indicate a statistically significant difference. All statistical analyses were performed using SPSS (Statistical Package for the Social Sciences) software, version 21.0.
