# Supplementary material for: TBC1D25 Regulates Cardiac Remodeling Through TAK1 Signaling Pathway
Source: Int J Biol Sci. 2020 Feb 21;16(8):1335–48. doi: 10.7150/ijbs.41130 (PMC7085222; doi:10.7150/ijbs.41130)
Supplement: Supplementary file 1 — Supplementary tables. [file ijbsv16p1335s1.pdf]

TBC1D25 Regulates Cardiac Remodeling Through TAK1 Signaling Pathway

Sen Guo[1*], Yuan Liu[1*], Lu Gao[1*], Fankai Xiao[2], Jihong Shen[3], Shiying Xing[4], Fan Yang[1], Wencai Zhang[1], Qiangwei Shi[1], Yan Li[1#], Luosha Zhao[1#]

[1] Department of Cardiology, The First Affiliated Hospital of Zhengzhou University, No.1 Jianshe East Road, Zhengzhou, China

[2] Henan Key Laboratory for Esophageal Cancer Research, the First Affiliated Hospital of Zhengzhou University

[3] Department of Electrocardiogram, The Second Affiliated Hospital of Zhengzhou University, No.2 Jingba Road, Zhengzhou, China

[4] Department of Cardiology, The First Affiliated Hospital, and College of Clinical Medicine of Henan University of Science and Technology, Luoyang, China.

[*] These authors contributed equally to this work.

[#] **Corresponding authors at:** Luosha Zhao, Tel: 86-0371-67967622; Fax: 86-0371-67967626; E-mail: zlszzu@126.com; Yan Li, Tel: 86-0371-66271072; Fax: 86-0371-66271073; E-mail: sophialiyan1987@126.com.

Supplementary table 1. The primer pairs used in this study

Gene name	Forward primer (mouse)	Reverse primer (mouse)
ANP	TCGGAGCCTACGAAGATCCA	TTCGGTACCGGAAGCTGTTG
BNP	GAAGGACCAAGGCCTCACAA	TTCAGTGCGTTACAGCCCAA
MYH7	CAACCTGTCCAAGTTCCGCA	TACTCCTCATTCAGGCCCTTG
Collagen Iα	TGCTAACGTGGTTCGTGACCGT	ACATCTTGAGGTCGCGGCATGT
Collagen III	ACGTAAGCACTGGTGGACAG	CCGGCTGGAAAGAAGTCTGA
CTGF	TGACCCCTGCGACCCACA	TACACCGACCCACCGAAGACACAG
GAPDH	ACTCCACTCACGGCAAATTC	TCTCCATGGTGGTGAAGACA

Supplementary table 2. The antibodies used in this study

Anbibody	Manufacturer	Catalogue number	Source of species	Dilution
TBC1D25	Boster	A11691	rabbit	1:500
ANP	Abclonal	A1609	rabbit	1:1000
p-TAK1	CST	4531	rabbit	1:1000
TAK1	CST	5206	rabbit	1:1000
p-ERK	CST	4370	rabbit	1:1000
ERK	CST	4695	rabbit	1:1000
P-JNK	CST	4668	rabbit	1:1000
JNK	CST	9252	rabbit	1:1000
p-p38	CST	4511	rabbit	1:1000
p38	CST	9212	rabbit	1:1000
p-p65	CST	3033	rabbit	1:1000
p65	CST	8242	rabbit	1:1000
Flag	MBL	M185	mouse	1:2000
HA	MBL	M180-3	mouse	1:2000
GAPDH	CST	2118	rabbit	1:5000
